# Synthesis of *N*-Glycolylneuraminic Acid (Neu5Gc) and Its Glycosides

**DOI:** 10.3389/fimmu.2019.02004

**Published:** 2019-08-28

**Authors:** Anoopjit Singh Kooner, Hai Yu, Xi Chen

**Affiliations:** Department of Chemistry, University of California, Davis, Davis, CA, United States

**Keywords:** sialic acid, sialoside, Neu5Gc, chemical synthesis, chemoenzymatic synthesis

## Abstract

Sialic acids constitute a family of negatively charged structurally diverse monosaccharides that are commonly presented on the termini of glycans in higher animals and some microorganisms. In addition to *N*-acetylneuraminic acid (Neu5Ac), *N*-glycolyl neuraminic acid (Neu5Gc) is among the most common sialic acid forms in nature. Nevertheless, unlike most animals, human cells loss the ability to synthesize Neu5Gc although Neu5Gc-containing glycoconjugates have been found on human cancer cells and in various human tissues due to dietary incorporation of Neu5Gc. Some pathogenic bacteria also produce Neu5Ac and the corresponding glycoconjugates but Neu5Gc-producing bacteria have yet to be found. In addition to Neu5Gc, more than 20 Neu5Gc derivatives have been found in non-human vertebrates. To explore the biological roles of Neu5Gc and its naturally occurring derivatives as well as the corresponding glycans and glycoconjugates, various chemical and enzymatic synthetic methods have been developed to obtain a vast array of glycosides containing Neu5Gc and/or its derivatives. Here we provide an overview on various synthetic methods that have been developed. Among these, the application of highly efficient one-pot multienzyme (OPME) sialylation systems in synthesizing compounds containing Neu5Gc and derivatives has been proven as a powerful strategy.

**Graphical Abstract:**
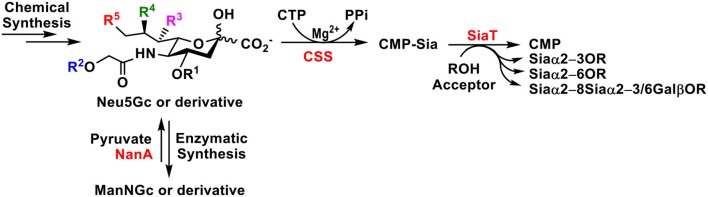
Chemical and chemoenzymatic synthetic methods for Neu5Gc and Neu5Gc-sialosides are reviewed. One-pot multienzyme (OPME) chemoenzymatic strategy has advantages in accessing a large number of Neu5Gc-sialosides and their derivatives.

## Introduction

**Figure 1:**
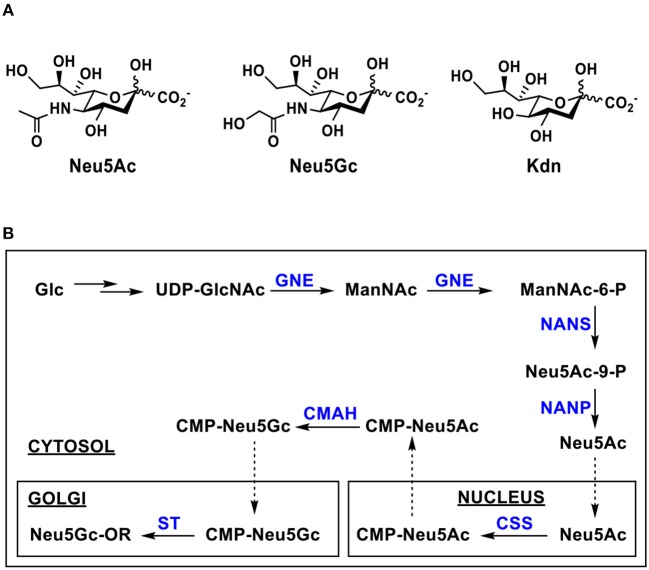
**(A)** Three basic forms of sialic acids including *N*-acetylneuraminic acid (Neu5Ac), *N*-glycolylneuraminic acid (Neu5Gc), and 3-deoxy-D-glycero-D-galacto-2-nonulosonic acid (Kdn); **(B)** Biosynthesis of Neu5Gc and its sialosides in eukaryotic cells. Enzymes and abbreviations: GNE, UDP-GlcNAc 2-epimerase/ManNAc-6-kinase; NANS, Neu5Ac-9-P synthetase; NANP, Neu5Ac-9-P phosphatase; CSS, CMP-sialic acid synthetase; CMAH, cytidine 5′-monophosphate-N-acetylneuraminic acid hydroxylase.

Sialic acids (Sias) are a family of negatively charged monosaccharides with a nine carbon backbone. More than 50 structurally distinct Sias have been found in nature (1–3), out of which more than 15 have been identified in human (4–6). They are commonly presented as the terminal monosaccharides of the carbohydrate moieties of glycoproteins and glycolipids on cell surface of deuterostome animals, in secreted glycans and glycoconjugates including those in the milk of mammals (2, 7–9). Some microorganisms including pathogenic bacteria also produce sialic acid and sialic acid-containing structures (7, 10). Three basic forms of sialic acids are *N*-acetylneuraminic acid (Neu5Ac), *N*-glycolylneuraminic acid (Neu5Gc), and 2-keto-3-deoxynonulosonic acid (Kdn) (Figure 1A) (1–3). In nature, sialic acid-containing oligosaccharides and glycoconjugates are formed mainly by sialyltransferase-catalyzed reactions transferring sialic acid from its activated sugar nucleotide, cytidine 5′-monophosphate-sialic acid (CMP-Sia), to suitable acceptors (11) although trans-sialidases have also been used by parasites and bacteria to harvest sialic acids from the hosts to decorate their own surface (12, 13). Neu5Ac is the most common form of sialic acids. Compared to Neu5Ac, Neu5Gc has an extra oxygen, presented as the hydroxyl in the *N*-glycolyl group at C-5.

The biosynthesis of Neu5Ac from uridine 5′-diphosphate *N*-acetylglucosamine (UDP-GlcNAc) in eukaryotic cells takes place in the cytosol by three enzymes. The first two committed steps are hydrolytic epimerization of UDP-GlcNAc to form *N*-acetylmannosamine (ManNAc) followed by phosphorylation to form ManNAc-6-P catalyzed by a single bifunctional enzyme UDP-GlcNAc 2-epimerase/ManNAc-6-kinase (GNE). Phosphoenolpyruvate is then condensed with ManNAc-6-P by Neu5Ac 9-phosphate synthase (NAPS) to produce Neu5Ac-9-P which is dephosphorylated to form Neu5Ac by Neu5Ac-9-phosphate phosphatase (NANP). The Neu5Ac synthesized in the cytosol is transferred into nucleus and used to form CMP-Neu5Ac, the activated form of Neu5Ac, by CMP-sialic acid synthetase (CSS). CMP-Neu5Gc formed in the cytosol from CMP-Neu5Ac by CMP-Neu5Ac hydroxylase (CMAH)-catalyzed reaction (11, 14–17) is transferred into Golgi and used by various sialyltransferases to form glycoconjugates which are secreted or expressed on cell surfaces (Figure 1B) (10, 18, 19). The *CMAH* gene is inactive in humans. Therefore, humans do not biosynthesize Neu5Gc-containing structures themselves (20, 21). New World monkeys were also shown to loss the function of Neu5Gc production due to an independent CMAH inactivation (22).

Regardless of CMAH inactivation in human, Neu5Gc has been found on the cell surface of human tumors and even in normal human tissues although at a lower amount (23, 24). Neu5Gc in human glycoconjugates comes likely from the consumption of animal-derived diets, such as red meat and animal milk (25–27). On the other hand, during infancy (around the age of 6 months) humans develop varying levels of polyclonal antibodies of IgG (28, 29), IgM, and IgA (30, 31) types against a diverse array of Neu5Gc-containing glycans (32–35). The mechanism of developing such anti-Neu5Gc antibodies early in the human life is unclear although incorporating dietary Neu5Gc by bacteria colonized in humans, such as non-typeable *Haemophilus influenzae* (NTHi) to form Neu5Gc-containing epitopes, such as cell surface lipooligosaccharides (LOS) is a likely source of the corresponding immunogens (32, 36, 37). So far, *de novo* synthesis of Neu5Gc and Neu5Gc-containing structures has not been demonstrated in bacteria. The presence of *CMAH*-like sequences has been found in the genomes of some bacteria but the activities of the corresponding enzymes have not been confirmed (38–40).

The presence of Neu5Gc-containing xeno-auto-antigens and anti-Neu5Gc xeno-autoantibodies in human (24) may lead to potential complications, such as chronic inflammation namely “xenosialitis” (41), atherosclerotic cardiovascular diseases, cancers, and autoimmune diseases (34, 38, 42–45). In addition, exposure to clinically used Neu5Gc-presenting animal-derived biotherapeutics (such as immunosuppressant rabbit anti-human thymocyte globulin, ATG) elicited anti-Neu5Gc antibodies (46, 47) with a profile that may be different from the “pre-existing” ones (28, 48). The biological consequences of this have not been revealed. A recent analysis showed that treating kidney transplant patients with ATG did not increase the risk of colon cancer (49). Neu5Gc has also been found on biodevices (such as bioprosthetic heart valves) which may affect their duration of function due to interaction with anti-Neu5Gc antibodies which can lead to calcification (50, 51). Furthermore, Neu5Gc in addition to α-Gal epitopes presented on animal tissues causes barriers for animal-to-human xenotransplantation (such as porcine skin xenografting and organ xenotransplantation) (52, 53).

Neu5Gc and its glycosides are important tools for profiling anti-Neu5Gc antibodies and sialic acid-binding proteins, understanding Neu5Gc-related immune responses, and designing potential therapeutics. To better understand their important roles, it is critical to obtain structurally defined glycans and glycoconjugates containing Neu5Gc or its derivatives.

**Figure 2:**
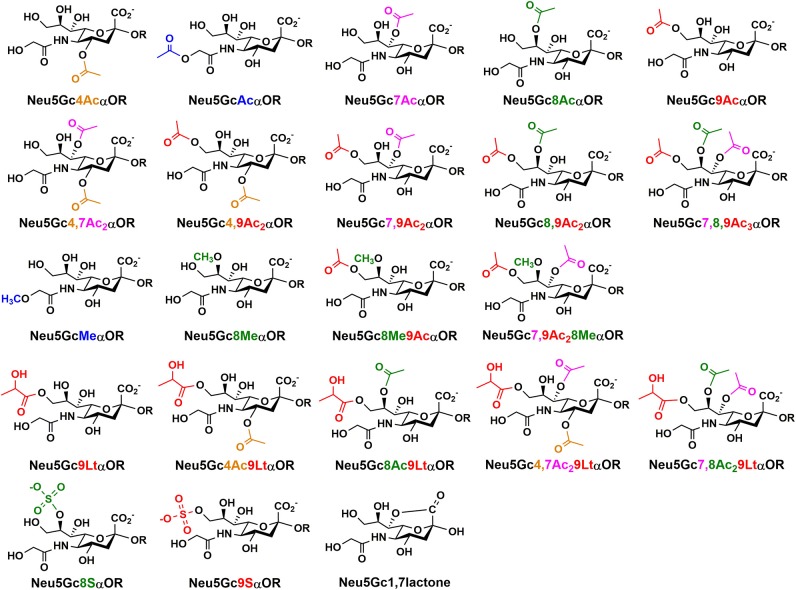
Naturally occurring Neu5Gc derivatives that have been identified.

Neu5Gc and derivatives have been found and can be isolated from natural sources including non-human mammals, some higher invertebrates, such as sea urchin, sea cucumber, and starfish (11, 23, 54, 55), as well as the surface of salmonid fish eggs (56). For example, Neu5Gc has been extracted from sea cucumber *Cucumaria echinate* in 99% purity. It constitutes about 85% of the total sialic acids in dry weight of Gumi (sea cucumber), and 23.6 mg was obtained from 135 g of fresh body weight (57). Neu5Gc-containing oligosaccharides have been reported in the milk of primates, domestic herbivores, pigs, lion, and leopard (58). So far, twenty-two Neu5Gc derivatives (Figure 2) have been reported (1, 3). These include mono-, di-, and tri-*O*-acetylation at C4, C5, C7, C8, and/or C9 positions in Neu5Gc as well as other modifications including *O*-methylation at C5- or C8-, *O*-lactylation at C9, or *O*-sulfation at C8 or C9 of Neu5Gc with or without *O*-acetylation. Neu5Gc1,7lactone has also been identified.

Neu5Gc and derivatives can link to other carbohydrate moieties with different sialyl linkages including α2–3- and α2–6-linked to galactose; α2–6-linked to *N*-acetylgalactosamine, *N*-acetylglucosamine, galactose or glucose; α2–8- and α2–9-linked to another Sia molecule; and α2–5-linked between polymers of Neu5Gc (7, 10, 59–61), adding diversity to sialic acid-containing compounds. The modification and linkage patterns of Sia play a pivotal role in many biochemical processes, such as cell signaling, cell-cell interaction, cellular adhesion, inflammation, fertilization, viral infection and malignancies, and regulation of apoptosis and proliferation (62, 63).

Numerous outstanding reports have been published describing the synthesis of sialic acids and sialoside. The focus, however, has been on Neu5Ac-containing compounds. The synthesis of Neu5Gc-containing glycans is attracting an increasing attention in recent years. This review provides an overview of various chemical and chemoenzymatic synthetic methods developed for the production of Neu5Gc and derivatives as well as the corresponding sialosides.

## Chemical and Chemoenzymatic Synthesis of Neu5Gc and Derivatives

Only a limited number of naturally occurring and non-natural Neu5Gc derivatives have been chemically or chemoenzymatically synthesized.

Neu5Gc was chemically synthesized from D-arabinose by the Wong group. The C5-acylamino group of Neu5Gc and a vinyl group were simultaneously introduced to D-arabinose by a modified Petasis coupling reaction. The vinyl group was then converted to γ-hydroxy-α-keto acid by a 1,3-dipolar cycloaddition reaction with *N*-*tert*-butyl nitrone followed by a base-catalyzed β elimination and hydrolysis to produce Neu5Gc in 22% overall yield (64).

*O*-Acetylation is the most frequent modification of Neu5Gc in nature. 9-*O*-Acetyl-Neu5Gc (Neu5Gc9Ac) has been found in bovine submandibular gland glycoprotein (65, 66). On the other hand, 4-*O*-acetyl-Neu5Gc (Neu4Ac5Gc) has been found in horse glycoproteins (61), α2–8-linked polysialic acids on glycoproteins from unfertilized kokanee salmon egg (67), the serum of guinea pigs (68), and gangliosides in human colon cancer tissues (69). Both Neu5Gc9Ac and Neu4Ac5Gc have been successfully synthesized. The use of orthoester intermediates is a very efficient method for producing various 9-*O*-acyl derivatives of Neu5Gc. Highly regioselective acylation at C9-hydroxyl of Neu5Gc was achieved by the treatment of Neu5Gc with a trimethyl orthoacetate in the presence of a catalytic amount of *p*-toluenesulfonic acid (*p*-TsOH) to form Neu5Gc9Ac in 90% yield. A similar strategy was applied for the synthesis of non-natural derivatives of Neu5Gc including 9-*O*-butyroyl-Neu5Gc and 9-*O*-benzoyl-Neu5Gc in 88 and 70% yields, respectively (70). On the other hand, Neu4Ac5Gc was synthesized using an efficient chemoenzymatic approach involving a sialic acid aldolase-catalyzed reaction from D-mannosamine (ManNH_2_) acylated with a benzyl protected *N*-glycolyl group. The obtained *N*-(2-benzyloxyacetyl)-D-mannosamine was enzymatically converted to a Neu5Gc derivative in a quantitative yield by recombinant *Pasteurella multocida* sialic acid aldolase (PmNanA) (71). Following a number of selective protection strategies, 4-hydroxyl group was selectively acetylated. The desired 4-OAc-Neu5Gc was obtained in an overall yield of 46% after de-protection of other hydroxyl groups (72).

In nature, the major function of sialic acid aldolases is to break down sialic acids, such as Neu5Ac to form 6-carbon amino sugar *N*-acetylmannosamine (ManNAc) and a three-carbon metabolite pyruvic acid. Nevertheless, they are capable of catalyzing the reversed reaction and have been used as synthetically useful enzymes for the formation of sialic acids and derivatives. Sialic acid aldolase-catalyzed reactions can be a general and highly efficient approach for chemoenzymatic synthesis of a diverse array of Neu5Gc and derivatives from the corresponding *N*-glycolylmannosamine (ManNGc) and derivatives. PmNanA was found to have a better expression level and more promiscuous substrate specificity than the more commonly used *Escherichia coli* sialic acid aldolase (EcNanA) in catalyzing the formation of sialic acids and derivatives (71). Both enzymes have been used for chemoenzymatic synthesis of Neu5Gc and derivatives. For the synthesis of Neu5Gc from ManNGc by sialic acid aldolase-catalyzed reaction, ManNGc could be obtained by chemical synthesis from D-mannosamine (ManNH_2_) (73, 74) or D-glucose (75), or by alkaline epimerization of *N*-acetylglucosamine (GlcNAc) (76). For the synthesis of ManNGc from ManNH_2_, the *N*-glycol group could be installed using commercially available inexpensive acetoxyacetyl chloride followed by de-*O*-acetylation by hydrolysis under a basic condition. However, it was found that ManNGc could be epimerized to form *N*-glycolylglucosamine (GlcNGc) under even mild basic conditions. *Aspergillus niger* lipase (Amano A) was found to be efficient in de-*O*-acetylation without the problem of epimerization (76). Installing the *N*-glycol group using *N*-succinimidyl glycolate (74) or 2-(benzyloxy)acetyl chloride followed by hydrogenation (77) could also avoid the complication of epimerization. The Neu5Gc formed went through additional chemical reactions for the synthesis of *N*-glycolyl-2,3-dehydro-2-deoxyneuraminic acid (Neu5Gc2en) (78), a transition state analog inhibitor of some sialidases. Together with *N*-acetyl-2,3-dehydro-2-deoxyneuraminic acid (Neu5Ac2en), they have been found to be effective in protecting mice from bacteria sepsis in a CD24/SiglecG-dependent manner (79) in a cecal ligation and puncture (CLP) mouse model (80). The protection was improved by combining the use of Neu5Ac2en and Neu5Gc2en with antibiotic treatment (79). In addition, Neu5Gc2en alone was effective in protecting mice from endotoxemia by inhibiting mouse sialidase NEU1 expressed on cell surface upon lipopolysaccharide (LPS) stimulation (81).

**Figure 3:**
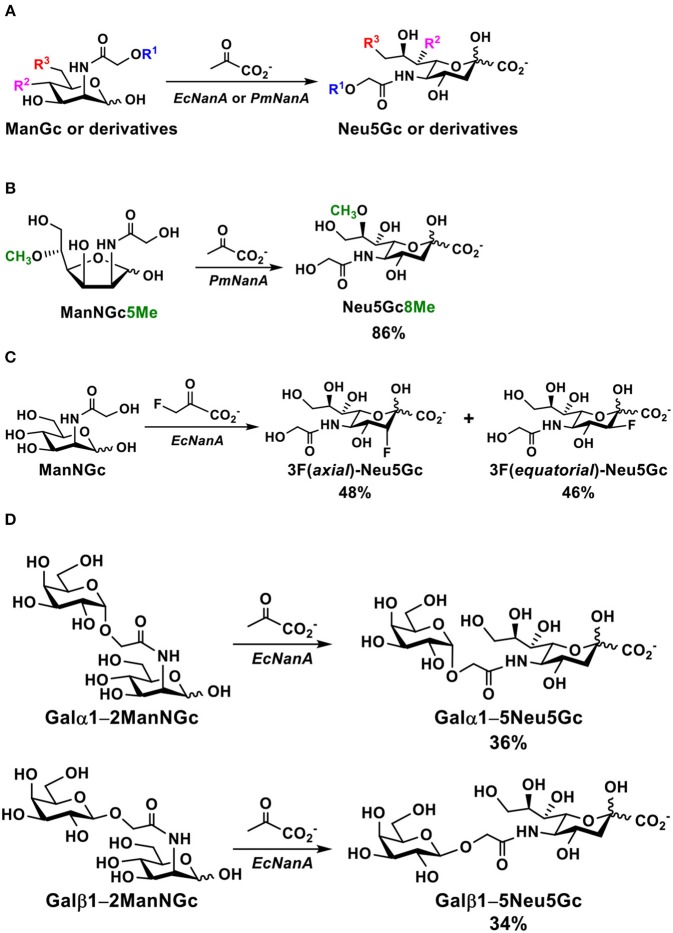
**(A)** A general chemoenzymatic synthetic strategy of sialic acid aldolase (EcNanA or PmNanA)-catalyzed synthesis of Neu5Gc and derivatives containing modifications at C5, C7, and/or C9 from ManNGc and derivatives, **(B)** PmNanA-catalyzed synthesis of Neu5Gc8Me, **(C)** EcNanA-catalyzed synthesis of 3-fluoro-Neu5Gc, and **(D)** EcNanA-catalyzed synthesis of disaccharides containing Neu5Gc at the reducing end.

The sialic acid aldolase-catalyzed reactions can also be used to synthesize naturally occurring and non-natural derivatives of Neu5Gc. As shown in Figure 3A, Neu5Gc derivatives with C5 and/or C9-modifications have been synthesized by sialic acid aldolase-catalyzed reactions from C2- and/or C6-substituted ManNGc derivatives as their 6-carbon sugar precursors (74, 82–87). Naturally occurring 8-*O*-methyl Neu5Gc (Neu5Gc8Me) (Figure 3B) was also synthesized from chemically synthesized 5-*O*-methyl ManNGc (ManNGc5Me) by a PmNanA-catalyzed reaction. A good yield of 86% was achieved using five equivalents of sodium pyruvate in Tris-HCl buffer (100 mM, pH 7.5) at 37°C for 24 h followed by the combination of anion exchange chromatography and gel filtration column purification (88).

EcNanA-catalyzed aldol addition of ManNGc and 3-fluoro-pyruvate resulted in a mixture of 3F(*equatorial*)Neu5Gc and 3F(*axial*)Neu5Gc with a ratio of close to 1:1. They were readily separated by a simple flash chromatography (Figure 3C) (89).

Disaccharides with a ManNGc at the reducing end could also be suitable substrates for EcNanA. Two chemically synthesized disaccharides Galα1–2ManNGc and Galβ1–2ManNGc were used as the substrates for EcNanA for the synthesis of the corresponding disaccharides Galα1–5Neu5Gc and Galβ1–5Neu5Gc in 36 and 34% yields, respectively (Figure 3D) (85).

## Synthesis of Simple Glycosides of Neu5Gc

**Figure 4:**
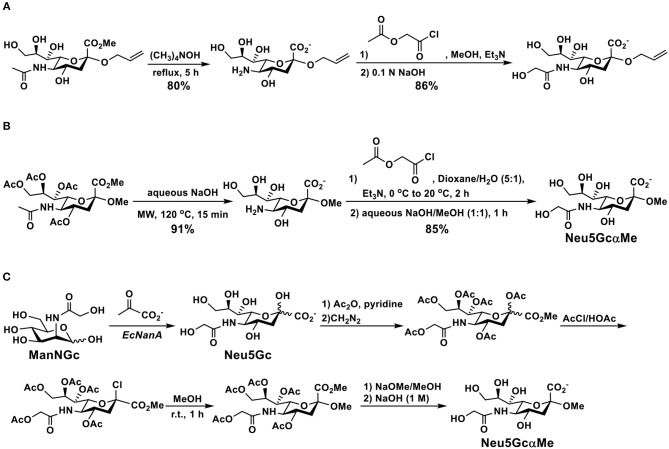
Synthesis of Neu5Gc-glycosides by de-*N*-acetylation of Neu5Ac-glycoside derivatives without **(A)** or with **(B)** a microwave-assisted process and **(C)** by glycosylation of protected Neu5Gc formed enzymatically from ManNGc and pyruvate.

Simple glycosides of Neu5Gc have been synthesized from the corresponding Neu5Ac derivatives by directly de-*N*-acetylating the *N*-acetyl group of Neu5Ac under a strong basic condition followed by acylation and deprotection. For example, as shown in Figure 4A, the *N*-acetyl group in the carboxyl protected allyl α-Neu5Ac-glycoside was removed to produce the free amino group in 80% yield by refluxing in tetramethylammonium hydroxide. Acylation with acetoxyacetyl chloride followed by hydrolysis of the ester produced the desired allyl α-Neu5Gc-glycoside (90). An improved microwave-assisted de-*N*-acetylation process was also reported (91). In this case, fully protected methyl α-Neu5Ac glycoside was treated with 2.0 M of NaOH under an optimized microwave irradiation condition (15 min at 120°C at a maximum power of 100 W) produced the desired 5-amino derivative in 91% yield. The resulting compound was then converted to the target methyl α-Neu5Gc glycoside (Neu5GcαOMe) by reacting with acetoxyacetyl chloride, followed by de-*O*-acetylation (Figure 4B). The same method was applied successfully for the formation of Neu5Gc2en from per-acetylated Neu5Ac2en methyl carboxylate as well as the production of poly-Neu5Gc from the corresponding α2–8-linked homopolymer of Neu5Ac (91).

An 9-azido derivative of Neu5Gc2en (Neu5Gc9N_3_2en) was also chemically synthesized from Neu5Ac9N_3_2en by substituting the 9-hydroxyl group with an azido group followed by replacing the -NHAc moiety with *N*-glycolyl group (92).

An alternative strategy for the synthesis of Neu5GcαOMe (Figure 4C) involved enzymatic formation of Neu5Gc from ManNGc using an EcNanA-catalyzed reaction. Protection of Neu5Gc, followed by activation, glycosylation, and deprotection led to the formation of the desired Neu5GcαOMe which was used for ELISA inhibition assays and for purifying anti-Neu5Gc antibodies from human sera (33).

## Chemical Synthesis of Neu5Gc-Containing Oligosaccharides

Several chemical glycosylation methods have been developed for the synthesis of Neu5Gc-containing oligosaccharides. The following discussion will be focused on different types of glycosyl donors used.

### Glycosyl Chloride Donors

A glycosyl chloride donor was used for synthesizing Neu5Acα2–5Neu5Gc disaccharide which contained a sialyl α2–5-Neu5Gc linkage similar to that found in the poly(-5Neu5Gcα2-) structure on the jelly coat of sea urchin eggs (93). The strategy involved the formation of an allyl glycoside of protected Neu5Ac as an important intermediate which went through oxidative cleavage of the C = C double bond in the allyl group (94) to form a protected Neu5Ac glycoside with a carboxymethoxy aglycone. Most recently, a similar strategy using protected Neu5Gc allyl glycoside donor was applied in the synthesis of Neu5Gcα2–5Neu5Gc disaccharide building block for the formation of a tetrasaccharide capped with 9-*O*-sulfo-Neu5Gc (Neu5Gc9S) found on sea urchin egg surface proteins (95).

The same Neu5Ac glycosyl chloride donor was used for glycosylation with methyl glycolate. The glycosylated product was deprotected and de-*N*-acetylated to form an amino-containing intermediate which can be either protected by a fluorenylmethyloxycarbony (Fmoc) group at the amino group or by a methyl group on the carboxyl groups. The resulting compounds were coupled to form the amide bond, linking two sialic acid units together to produce the desired disaccharide containing a Neu5Gc residue (96). α2–5-Linked Neu5Gc oligomers for up to octasaccharide were also synthesized using a similar strategy by coupling carboxyl and amine protecting groups of sialic acid building blocks by amide formation (97).

An *O*-acetyl protected Neu5Gc glycosyl chloride donor was also used for the synthesis of Neu5Gcα2–3Galβ1–4Glc trisaccharide building block for the formation of Neu5Gc-GM3 ganglioside although with a low yield and a poor stereo-selectivity (98).

### Thioglycoside Donors

Sialyl thioglycoside donors have been widely applied in chemically formation of sialyl glycosidic bonds. A thioglycoside donor of Neu5Ac was used for the synthesis of Neu5Acα2–5Neu5Gc disaccharide found as the structural component of the jelly coat of sea urchin eggs. The strategy relied on the formation of a protected Neu5Ac glycoside with a carboxymethoxy aglycone which was readily coupled with the amino group of the protected neuraminic acid to form the desired amide bond in the disaccharide (93).

Thioglycoside donors of Neu5Gc have also been used for the synthesis of more complex Neu5Gc-containing sialosides. The Kiso group reported the synthesis of protected Neu5Gcα2–3GalβOMP disaccharide using *N*-2,2,2-trichloroethoxycarbonyl (Troc)-protected thiophenyl sialoside donor which was readily obtained from its corresponding *N*-acetyl derivative. Sialylation of a selectively protected galactoside acceptor led to the formation of sialyl disaccharide. Removal of the *N*-Troc group by zinc in acetic acid formed a free amino group which can be acylated with acetoxyacetyl chloride to produce the desired protected Neu5Gc-containing disaccharide (99). A similar strategy was used for the synthesis of a Neu5Gc8Me-containing tetrasaccharide building block of the pentasaccharide component, Neu5Gc8Meα2–3(Neu5Gc8Meα2–6)GalNAcβ1–3Galβ1–4Glc, in GAA-7 ganglioside (100). In addition to the use of acetoxyacetyl chloride as a reagent for introducing a protected glycolyl group to the amino group on neuraminic acid (Neu) residue for the formation of Neu5Gc, 1,3-dioxolan-2,4-dione (101) prepared from glycolic acid was also used for the formation of Neu5Gc-GM1 ganglioside directly from naturally more abundant Neu5Ac version of GM1 (102).

The Sato group used a *N*-Troc-protected thiophenyl sialoside donor for the synthesis of sialyllactoside component of ganglioside LL3 tetrasaccharide. The removal of the *N*-Troc group followed by conjugation with a protected Neu5Ac glycoside with a carboxymethoxy aglycone and deprotection steps formed the desired LL3 tetrasaccharide (103, 104).

The amino intermediate of the protected sialoside formed after the removal of the *N*-Troc group could be converted directly (99) to a 1,5-lactamized bicycle structure. Alternatively, *N*-trifluoroacetyl (*N*-TFA)-protected thiophenyl sialoside donor can also be used similarly for the formation of sialyl glycosides. The *N*-TFA group could be readily removed and the resulting amino group-containing intermediate could be converted to a 1,5-lactamized bicycle structure under mild basic conditions. The resulting intermediate could be selectively protected at C-9 of the sialic acid and used as a well-suited sialylation acceptor. A similar *N*-Troc and 8,9-acetal-protected thiotoluene sialoside donor was used for the synthesis of protected Neuα2–6GalαSer as the sialyl Tn disaccharide building block that was coupled with the pre-formed Neu5Gcα2–5Neu5Gc disaccharide component for the formation of the sea urchin egg surface Neu5Gc9S-capped tetrasaccharide (95).

Trisaccharide Neu5Gcα2–4Neu5Acα2–6Glc, a structural component of ganglioside HLG-2, was synthesized by the Kiso group by stereoselective coupling of *N*-Troc-protected thiophenyl Neu5Gc-sialoside donor with the pre-formed Siaα2–6Glc 1,5-lactamized disaccharide acceptor (105, 106). A similar strategy was used for the synthesis of Fucα1–4Neu5Acα2–5Neu5Gcα2–4Neu5Acα2–6Glc, a pentasaccharide component of HPG-7 ganglioside (107) and Fucα1–8Neu5Gcα2–4Neu5Acα2–6Glc, a tetrasaccharide components of ganglioside HPG-1 (108).

The Crich group reported the synthesis of Neu5Gc-containing oligosaccharides in high stereoselectivity by iterative one-pot route. A series of four trisaccharides were synthesized in one pot by coupling of a 5*N*-acetoxyacetimide-5*N*, 4*O*-oxazolidinone-protected adamantanyl thiosialoside donor with the first thiogalactosyl acceptor followed by addition of the second acceptor after 20 min (109).

The Nifant'ev group used an *N*-*tert*-butyloxycarbonyl (*N*-Boc) and *N*-acetyl (*N*-Ac) protected thiophenyl sialoside donor for the synthesis of 3-aminopropyl glycoside of Neu5Gcα2–6LacNAc from *N*-acetyllactosamine (LacNAc) 4′,6′-diol acceptor (30). A glycosylation yield of 84% with 1.3:1 (α:β) selectivity was achieved. Removal of the *N*-acetyl and *N*-Boc groups followed by *N*-acylation and subsequent deprotection steps formed the desired trisaccharide. A similar strategy was used for the synthesis of 3-aminopropyl glycoside of Neu5Gcα2–3LacNAc using LacNAc 2′,3′,4′-triol acceptor (110).

Instead of installing *N*-glycolyl group after the formation of sialyl glycosidic bond, properly protected Neu5Gc thioglycoside donors could be directly used for glycosylation. For example, an acetyl-protected thiophenyl Neu5Gc-glycoside donor was used directly with α-selectivity and good sialylation yields for the synthesis of Neu5Gc-containing glycosides including sialyl Lewis × pentasaccharyl ganglioside analog (111) and α2–3-sialyl lactotetraose and neolactotetraose derivatives (112). A thiophenyl Neu5Gc-glycoside donor was also successfully used for the synthesis of Neu5Gcα2–6GalOMP disaccharide and its derivative Neu5Gc9N_3_α2–6GalOMP containing a 9-azido-9-deoxy-Neu5Gc residue. The 9-azido group of the latter was converted to an amino group and the resulting compound was used to generate a library of 9-*N*-acylated derivatives of Neu5Gc-sialosides. Some of the compounds were low-micromolar inhibitors of CD22 (or Siglec-2), a well-known B cell-specific sialic acid-binding immunoglobulin-like lectin (113). The same strategy was used to synthesize a similar class of sialosides with different aglycons as improved CD22 inhibitors with up to nanomolar potency (114, 115). In addition to protected thiophenyl Neu5Gc-glycoside donors, a benzyl-protected thiomethyl Neu5Gc-glycoside donor was developed and used for the synthesis of Neu5Gc-containing trisaccharides with 55–63% yields with α-selectivity (116) and a sea cucumber disaccharyl ganglioside analog (117).

### Phosphite Donors

**Figure 5:**
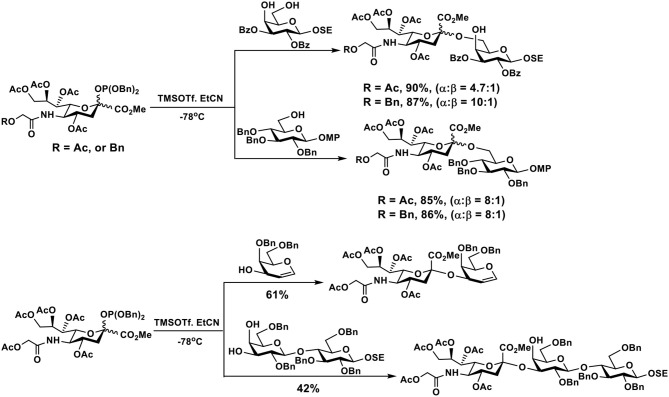
Efficient synthesis of Neu5Gc-glycosides using phosphite donors in good yields and α-selectivity.

Phosphite donors of Neu5Gc are considered to be more reactive than thioglycoside donors. They were used for the synthesis of Neu5Gc-glycosides in propionitrile at −78°C in good yields and α-selectivity (Figure 5) (118).

### Trichloroacetimidate Donors

Trichloroacetimidate donors are the most commonly used glycosyl donors. They have been used for the synthesis of complex Neu5Gc-containing sialosides. The Kiso group reported the first total synthesis of Neu5Gc8Me-containing ganglioside GAA-7 which showed neuritogenic activity. The strategy involved the assembly of the ceramide moiety by Witting, Grignard, and amide formation reactions. Stereoselective β-glycosylation with a glucosyl trichloroacetimidate donor produced a glucosyl ceramide (GlcβCer) cassette which was readily coupled with the protected Neu5Gc-containing tetrasaccharyl trichloroacetimidate donor to form the protected ganglioside. Global deprotection produced GAA-7, a pentasaccharyl β-ceramide Neu5Gc8Meα2–3(Neu5Gc8Meα2–6)GalNAcβ1–3Galβ1–4GlcβCer (119). Protected Neu5Gc-containing disaccharyl trichloroacetimidate donors have also been used for the synthesis of Neu5Gc-containing glycans of lacto- and neolacto-series gangliosides. The reducing ends of these oligosaccharides were further modified by 2-(tetradecyl)hexadecanol to form glycolipid mimics of ceramide-containing gangliosides (120).

### *N*-Phenyltrifluoroacetimidate Donors

**Figure 6:**
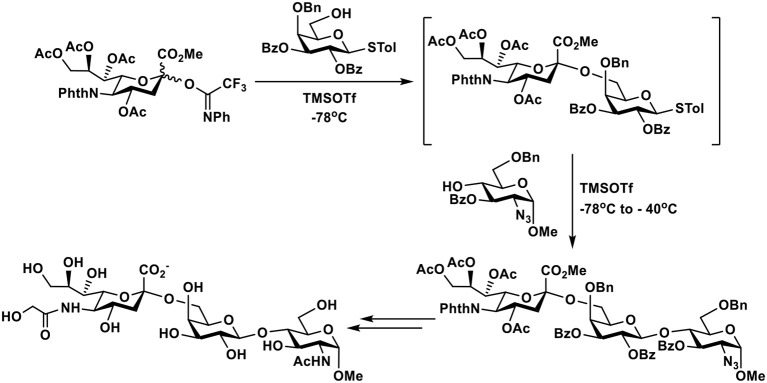
An example of one-pot chemical synthesis of Neu5Gc-containing trisaccharides using 5-*N*-phthaloyl group protected *N*-phenyltrifluoroacetimidate sialyl donor.

*N*-Phenyltrifluoroacetimidate (121) sialyl donors were designed to improve their reactivity for glycosylation. The feature was combined with 5-*N*-phthaloyl group protection of the sialyl donors to favor α-sialyl isomer formation (122, 123) and allow their suitability for one-pot procedures (124). As shown in Figure 6, desired α-sialoside was stereoselectively synthesized using this donor. The synthesized α-sialoside was further coupled to another acceptor in one-pot to synthesize trisaccharides with various internal disaccharide units. The 5-*N*-phthaloyl group on sialic acid of trisaccharides was readily removed, acylated, and deprotected to form the *N*-glycolyl group in Neu5Gc (124).

## Chemoenzymatic Synthesis of Neu5Gc-Containing Oligosaccharides

Sialyltransferase-catalyzed glycosylation can be considered as the most efficient approach for the production of sialic acid-containing structures. The strategy offers great advantages, including high regioselectivity and stereoselectivity for the formation of sialyl linkages as well as mild reaction condition in aqueous solutions, etc. (2, 10). The increasing availability of substrate promiscuous sialyltransferases in large amounts makes the strategy practical even for large-scale synthesis. As the sugar nucleotide donor, CMP-Neu5Gc, for sialyltransferase-catalyzed synthesis of Neu5Gc-glycosides is not commercially available, additional enzymes including CMP-sialic acid synthetases (CSSs) with or without sialic acid aldolases are commonly used. Although biosynthetically CMP-Neu5Gc is directly synthesized from CMP-Neu5Ac by CMAH-catalyzed hydroxylation, Neu5Gc is a well-tolerated substrate for CSSs from bacterial sources including those from *Neisseria meningitidis* (NmCSS), *Escherichia coli* (EcCSS), *Streptococcus agalactiae* serotype V (SaVCSS), *Pasteurella multocida* strain P-1059 (PmCSS), *Haemophillus ducreyi* (HdCSS), and *Clostridium thermocellum* (CtCSS) (74, 125, 126). Among these, NmCSS with a high expression level, a high specific activity, and substrate promiscuity is an excellent choice for chemoenzymatic synthesis of sialosides with or without sialic acid modifications (125).

**Figure 7:**
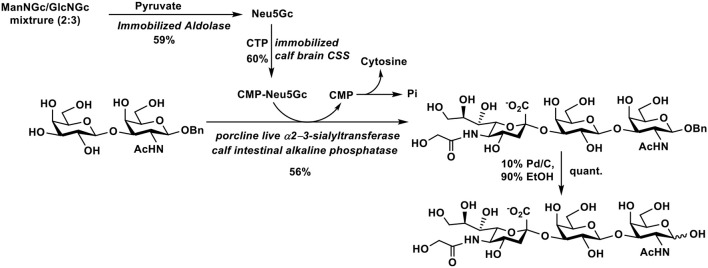
Chemoenzymatic synthesis of Neu5Gcα2–3Galβ1–3GalNAc.

Starting from pyruvate and a mixture of ManNGc and GlcNGc, chemoenzymatic synthesis of trisaccharide Neu5Gcα2–3Galβ1–3GalNAc, which has been found in porcine submaxillary mucin, was achieved (127). As shown in Figure 7, Neu5Gc was synthesized in 59% yield using an immobilized sialic acid aldolase. It was used for the formation of CMP-Neu5Gc using an immobilized calf brain CMP-sialic acid synthetase in 60% yield. Sialylation of Galβ1–3GalNAcβOBn was carried out by a porcine liver α2–3-sialyltransferase-catalyzed reaction using CMP-Neu5Gc as donor. Deprotection by catalytic hydrogenation produced the target trisaccharide Neu5Gcα2–3Galβ1–3GalNAc in 56% yield.

As reaction conditions for sialic acid aldolase, CSS, and sialyltransferase are compatible, they can be mixed together in one-pot with ManNGc, pyruvate, CTP, and a sialyltransferase acceptor for the synthesis of target Neu5Gc-glycosides. Such one-pot multienzyme (OPME) sialylation reactions (82, 84, 86, 128) are highly efficient for chemoenzymatic synthesis of a large library of Neu5Gc-glycosides containing different sialyl linkages and various internal glycans. Sialosides containing modified Neu5Gc forms can also be produced by this strategy.

**Figure 8:**
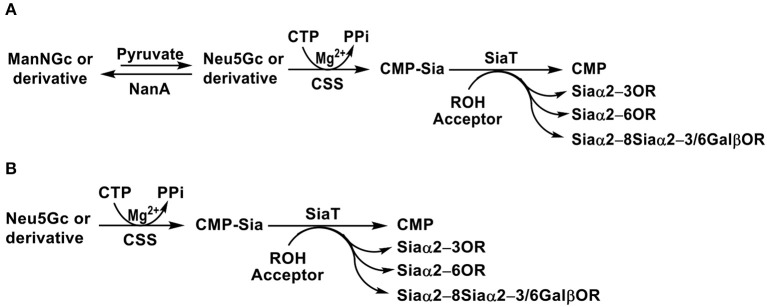
Synthesis of sialosides containing Neu5Gc or derivative using one-pot multienzyme (OPME) sialylation systems containing **(A)** three enzymes including sialic acid aldolase (NanA), CMP-sialic acid synthetase (CSS), and sialyltransferase (SiaT) or **(B)** two enzymes including CSS and SiaT.

As shown in Figure 8A, in the OPME reaction containing a sialic acid aldolase, a CSS, and a sialyltransferase, chemically synthesized ManNGc or derivative is enzymatically converted to Neu5Gc or derivative by the sialic acid aldolase. Activation of the formed Neu5Gc or derivative to CMP-Neu5Gc or derivative by CSS followed by sialylation led to the production of the desired sialoside containing Neu5Gc or derivative. Both sialic acid and CMP-sialic acid are generated *in situ* and do not need to be purified.

If Neu5Gc and derivatives are available, OPME reaction containing a CSS and a sialyltransferase without the presence of a sialic acid aldolase (Figure 8B) is sufficient to produce sialosides containing Neu5Gc or derivatives. The strategy is particularly suited for sialosides containing a Neu5Gc derivative that cannot be directly obtained by a sialic acid aldolase-catalyzed reaction, such as Neu4Ac5Gc (72). The method was also used for synthesizing sialosides containing 3F(*equatorial*)-Neu5Gc or 3F(*axial*)-Neu5Gc. In this case, 3F(*equatorial*)-Neu5Gc, and 3F(*axial*)-Neu5Gc were pre-synthesized from ManNGc and 3-fluoro-pyruvate by EcNanA-catalyzed reaction and purified before being subjected to OPME sialylation reactions.

Using efficient OPME sialyltransferase systems with two- or three-enzymes (Figure 8), a diverse array of sialosides including glycosphingolipid glycans, sialylated types 1–5 glycans, and sialyl Tn, containing Neu5Gc (Table 1) as well as sialosides containing different Neu5Gc derivatives including 3F-Neu5Gc, Neu4Ac5Gc, Neu5Gc9Ac, Neu5GcMe, or Neu5GcAc (Table 2) have been synthesized. The obtained compounds have been used to construct sialyl glycan microarrays (28, 33, 34, 42, 83, 150–156), sialoside-protein conjugates (148), and sialidase substrate specificity studies (131, 157–159). Among bacterial sialyltransferases used, *Pasteurella multocida* sialyltransferase 1 (PmST1) (84) and its single mutant PmST1 M144D with decreased donor hydrolysis and sialidase activities (141) were broadly applied for the synthesis α2–3-linked sialyl oligosaccharides containing Neu5Gc, 3F-Neu5Gc, Neu5Gc9Ac, Neu5GcMe, or Neu5GcAc (77, 83, 84, 89, 157, 160). For synthesizing α2–3-sialyl oligosaccharides containing Neu4Ac5Gc, however, only *Pasteurella multocida* sialyltransferase 3 (PmST3) (161) was found to be a suitable enzyme (72). PmST3 was also well-suited for the synthesis of α2–3-linked Neu5Gc-containing sialyl glycopeptides (162). *Photobacterium damselae* α2–6-sialyltransferase (Pd2,6ST) (82), *Photobacterium* species α2–6-sialyltransferase (Psp2,6ST) (87) and its single mutant with improved expression level and slightly enhanced activity Psp2,6ST A366G (163) were used for synthesizing α2–6-linked sialosides containing Neu5Gc, 3F-Neu5Gc, Neu5Gc9Ac, Neu5GcMe, or Neu5GcAc (82, 83, 89, 157, 160). Psp2,6ST was well-suited for the synthesis of sialyl Tn-antigens (Sia α2–6GalNAcαOR) (87). *Campylobacter jejuni* sialyltransferase CstII (CjCstII) (164) was found to be an efficient sialyltransferase for the synthesis of a diverse array of Neu5Gc-containing α2–8-linked sialosides (77, 86, 131). For synthesizing sialosides containing Neu5Gc or its stable analogs, such as 3F-Neu5Gc and Neu5GcMe, the pH of the OPME reactions was controlled at 8.5 to allow highly efficient catalysis by all enzymes involved in the reactions. For synthesizing sialosides containing base-labile groups, such as Neu4Ac5Gc, Neu5GcAc, or Neu5Gc9Ac, the pH of the OPME reactions was controlled at 7.0 to minimize de-*O*-acetylation during the reaction.

**Table 1:**
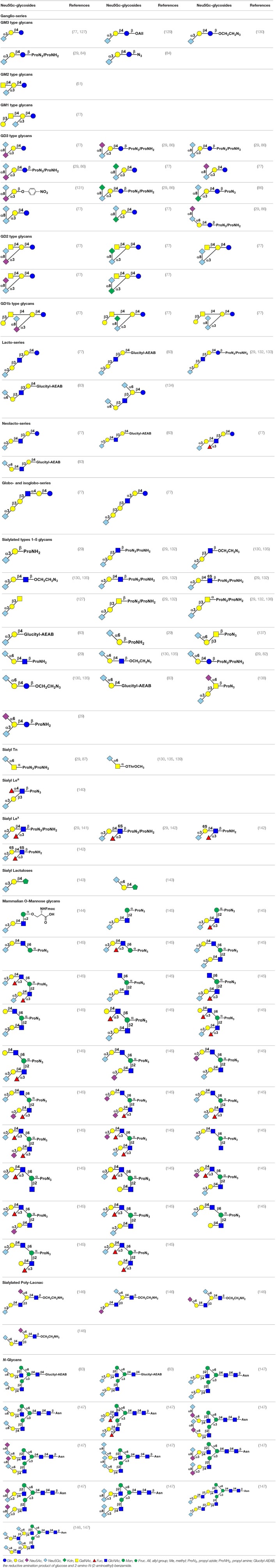
Chemoenzymatically synthesized Neu5Gc-containing glycans.

**Table 2:**
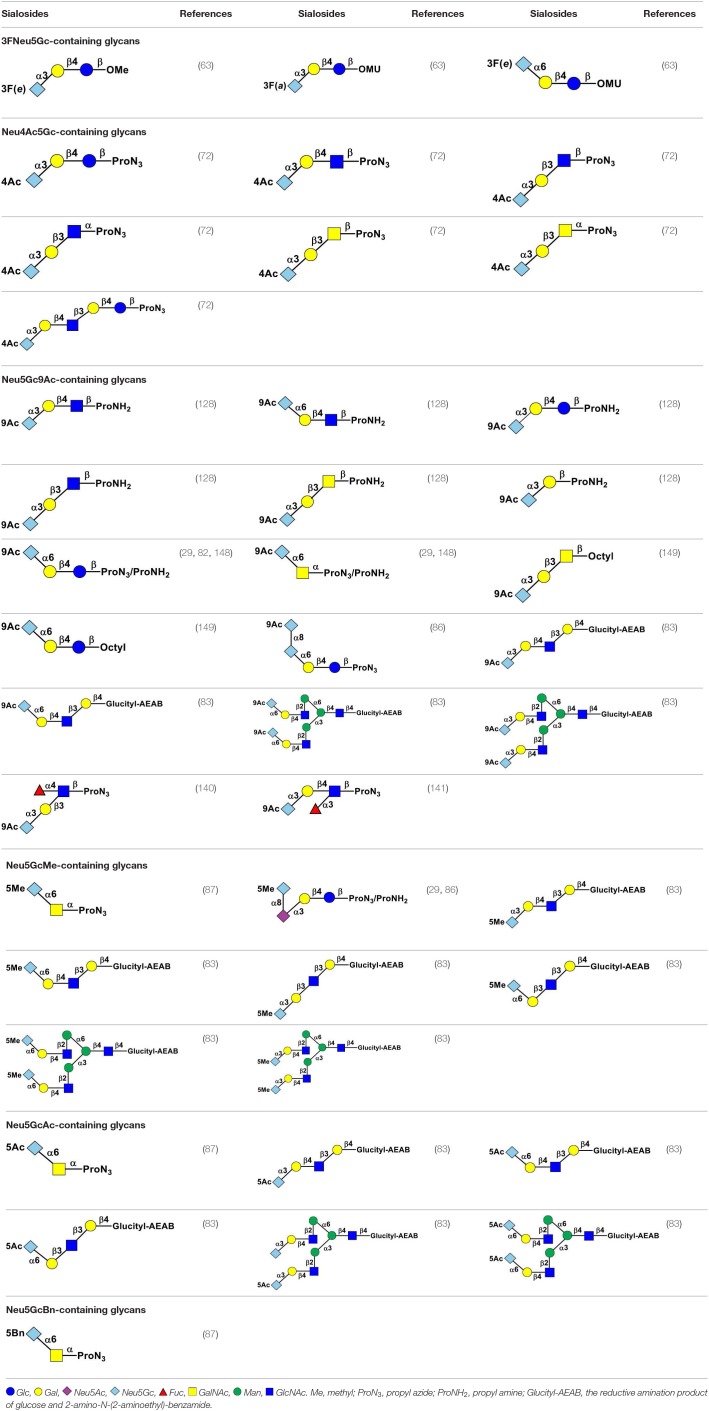
Chemoenzymatically synthesized sialosides containing 3FNeu5Gc, Neu4Ac5Gc, Neu5Gc9Ac, Neu5GcMe, Neu5GcAc, or Neu5GcBn.

Recently, the OPME α2–3-sialylation system containing PmNanA, NmCSS, and PmST1 M144D was coupled with *Streptococcus pneumoniae* sialidase SpNanC-catalyzed reaction for the formation of Neu5Gc2en from ManNGc, pyruvate, CTP, and lactose (165).

## Chemoenzymatic Synthesis of Neu5Gc-Containing Glycoconjugates

**Figure 9:**
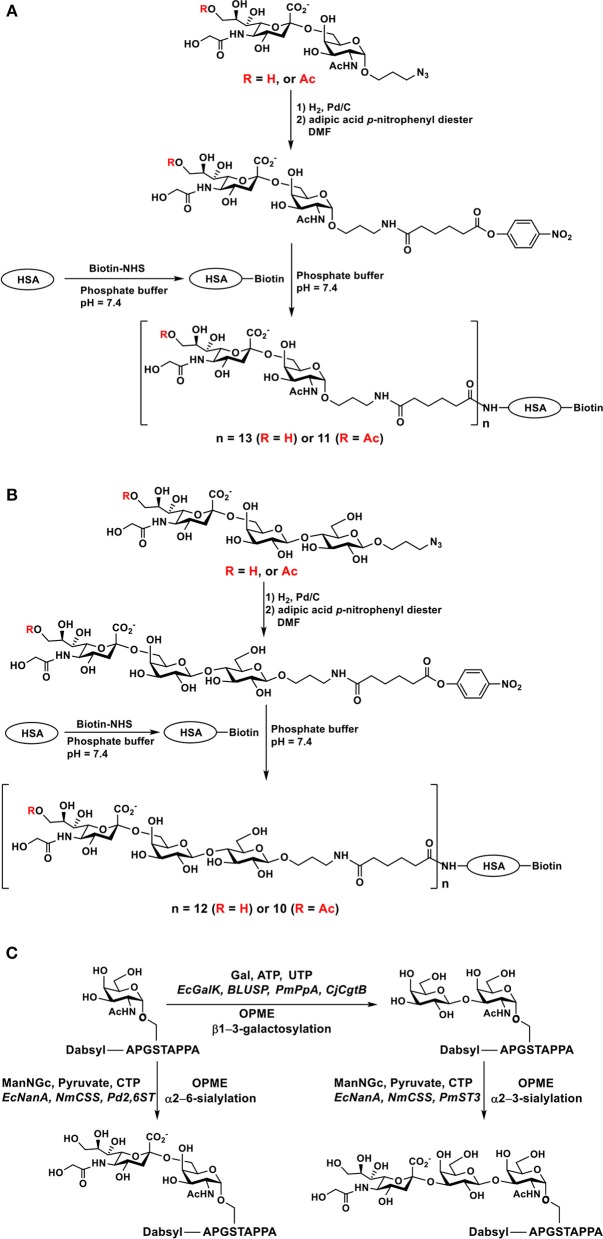
Synthesis of biotinylated human serum albumin-sialoglycoside conjugates containing Neu5Gc or Neu5Gc9Ac including **(A)** sTn epitopes, **(B)** sialyl lactoside, and **(C)** chemoenzymatic synthesis of dabsyl fluorophore-tagged glycopeptides including sTn, T, and ST-antigens containing Neu5Gc. Adopted and modified from Yu et al. (148) and Malekan et al. (162) with permission.

The alkyl azido aglycone in chemoenzymatically synthesized Neu5Gc-containing sialosides can be readily converted to an alkyl amino group by catalytic hydrogenation to allow convenient conjugation with *N*-hydroxysuccinimide-activated or epoxide-activated slide surface for generating glycan microarrays (34). It was also used to react with adipic acid *p*-nitrophenyl diester to form half-esters which were coupled to the amino group (e.g., in lysine residues) of biotinylated human (STn) antigens Neu5Gc/Neu5Gc9Acα2–6GalNAcαOR (Figure 9A) and sialyl lactosides Neu5Gcα2–6Galβ1–4GlcβOR (Figure 9B) containing Neu5Gc or Neu5Gc9Ac were successfully synthesized and used for ELISA inhibition studies (33, 148).

OPME chemoenzymatic sialylation reactions have also been used in the synthesis of sialyl-Tn-MUC1 and sialyl-T-MUC1 glycopeptides containing Neu5Gc (Figure 9C). *Pasteurella multocida* α2–3-sialyltransferase (PmST3), *Photobacterium damselae* α2–6-sialyltransferase (Pd2,6ST) *Neisseria meningitidis* CMP-sialic acid synthetase (NmCSS) and *E. coli* sialic acid aldolase are the enzymes used for OPME sialylation of glycoproteins (162).

Hidari et al. recently reported the synthesis of multivalent Neu5Gc-containing sialoglycopolypeptides. Treating the chemically synthesized Lac or LacNAc-carrying peptides as acceptors and CMP-Neu5Gc as the donor substrate, sialoglycopolypeptides with α2–3- and α2–6-sialyl linkages were obtained in the presence of ST3Gal III or ST6Gal I, respectively. They found that multivalent α2–3-linked Neu5Gc-ligands selectively inhibited hemagglutination mediated by influenza viruses with a strong inhibitory activity (166). Hernaiz et al. also reported that the enzymatic approach could be directly applied to sialylating lactose-carrying glycoclusters using α2–6-sialyltransferase from rat liver and CMP-Neu5Gc as the donor to produce Neu5Gc-containing glycoclusters (167).

## Conclusions and Perspective

Significant advances have been made in the synthesis of sialosides although the focus has been on those containing Neu5Ac, the most common sialic acid form. With the increasing recognition of the presence and the important functions of Neu5Gc and human anti-Neu5Gc xeno-autoantibodies, more attention has been and will be paid to the synthesis of sialosides containing Neu5Gc and its derivatives. Chemical synthetic methods developed for the formation of Neu5Ac-containing molecules can be extended to Neu5Gc counterparts with modifications. Chemoenzymatic methods using sialyltransferases have been recognized as efficient strategies for accessing challenging sialic acid-containing molecules including those containing Neu5Gc and derivatives. Among these, one-pot multienzyme (OPME) systems have been proven powerful tools. Large library of sialosides containing Neu5Gc and derivatives will become available for elucidating their biological roles and exploring their potential applications. These will be indispensable probes for profiling anti-Neu5Gc antibodies and investigating other Neu5Gc-binding proteins. Such information will help us to better understand the physiological and pathological roles of Neu5Gc and its binding partners. Combining sialidase-treatment and sialyltransferase-catalyzed re-sialylation with Neu5Gc or Neu5Ac will be a potentially efficient approach for generating glycoconjugates with a desired sialic acid form for improved therapeutic applications.

## Author Contributions

AK, HY, and XC searched the literature, read the papers, and wrote the manuscript.

### Conflict of Interest Statement

The authors declare that the research was conducted in the absence of any commercial or financial relationships that could be construed as a potential conflict of interest. The handling Editor declared a past co-authorship with one of the authors XC.
